# Occupational exposure and sinonasal cancer: a systematic review and meta-analysis

**DOI:** 10.1186/s12885-015-1042-2

**Published:** 2015-02-13

**Authors:** Alessandra Binazzi, Pierpaolo Ferrante, Alessandro Marinaccio

**Affiliations:** Italian National Workers’ Compensation Authority (INAIL) Department of Occupational and Environmental Medicine, Epidemiology and Hygiene – Unit of Occupational and Environmental Epidemiology, Via Stefano Gradi 55, 00143 Rome, Italy

**Keywords:** Sinonasal cancer, Occupational exposure, Epidemiology, Meta-analysis, Systematic review

## Abstract

**Background:**

Sinonasal cancer (SNC) has been related to occupational exposures, but the relative risk associated to specific jobs and/or carcinogen exposures other than wood and leather dust is generally based on small or inadequate sample sizes and the range of observed estimates is large. This paper is aimed at investigating such relationship through a systematic review of the literature followed by a meta-analysis of studies meeting specific inclusion criteria.

**Methods:**

Systematic search was made with PubMed, Google Scholar and Scopus engines using related keywords. Occupational exposures include wood and leather dust, formaldehyde, nickel and chromium compounds, textile industry, farming and construction. Meta-analysis of published studies after 1985 with a case-control or cohort design was performed, firstly using the fixed-effect model. Heterogeneity was assessed with the Q statistical test and quantified by the I^2^ index. When the heterogeneity hypothesis appeared relevant, the random-effect model was chosen. Sources of heterogeneity were explored using subgroup analyses.

**Results:**

Out of 63 reviewed articles, 28 (11 cohort, 17 case-control) were used in the meta-analysis. Heterogeneity among studies was observed and random-effects models were used. Exposure to wood dust results associated with SNC (RR_pooled_ = 5.91, 95% CI: 4.31-8.11 for the case-control studies and 1.61, 95% CI: 1.10-2.37 for the cohort studies), as well as to leather dust (11.89, 95% CI: 7.69-18.36). The strongest associations are with adenocarcinomas (29.43, 95% CI: 16.46-52.61 and 35.26, 95% CI: 20.62-60.28 respectively). An increased risk of SNC for exposures to formaldehyde (1.68, 95% CI: 1.37-2.06 for the case control and 1.09, 95% CI: 0.66-1.79 for the cohort studies), textile industry (2.03, 95% CI: 1.47-2.8), construction (1.62, 95% CI: 1.11-2.36) and nickel and chromium compounds (18.0, 95% CI: 14.55-22.27) was found. Subset analyses identified several sources of heterogeneity and an exposure-response relationship was suggested for wood dust (p = 0.001).

**Conclusions:**

By confirming the strength of association between occupational exposure to causal carcinogens and SNC risk, our results may provide indications to the occupational etiology of SNC (not only wood and leather dusts). Future studies could be focused on specific occupational groups to confirm causative agents and to define appropriate preventive measures.

**Electronic supplementary material:**

The online version of this article (doi:10.1186/s12885-015-1042-2) contains supplementary material, which is available to authorized users.

## Background

Sinonasal malignant neoplasms (ICD-10: C30-C31; ICD-9: 160) are rare tumors with annual incidence rates around 1 per 100,000 in most developed countries. They represent less than 1% of all neoplasms and less than 4% of those arising in the head and neck region [1-4]. The overall incidence in the US between 1973 and 2006 was estimated at 0.6 cases per 100,000 [5], while in Europe (1978-2002) was lower than 0.5 per 100,000 [6]. In Italy the incidence rates in the period 1998–2002 were estimated at 0.4-2.0 per 100,000 in men and 0.1-0.5 per 100,000 in women. There was a high variability across Italian regions with about 300 expected cases per year in the whole country [7].

A recent US analysis on sinonasal cancer (SNC) incidence and survival found that almost half of SNCs are localized to the nasal cavity (43.9%), most others originated in the maxillary (35.9%) or ethmoid (9.5%) sinus. These lesions were composed mostly of tumors of epithelial origin, including squamous cell carcinomas (SCC: 51.6%), adenocarcinomas (AC: 12.6%), esthesioneuroblastoma (ENB: 6.3%), and adenoid cystic carcinoma (ACC: 6.2%) [5]. A progress has been evidenced in outcome and survival during the last years, with average overall 5-year survival rates ranging from less than 30% in the 1960s to over 50% in the 2000s [5-9]. The low absolute risk in the general population associated with high relative risks for specific chemical exposures and occupational settings, has entitled SNC a ‘sentinel’ for monitoring occupational and environmental risk factors.

A number of substances and occupational circumstances causing or possibly causing SNC have been classified by IARC as Group 1 and 2A. Tobacco smoking and other life-style factors seem to play a minor role (if any) in the etiology of SNC [10,11]. Occupational exposures to wood and leather dust have been strongly associated with SNC [12-19]. Since the initial evidence, SNC risk among woodworkers has been investigated in many epidemiologic studies [20-23]. Wooden furniture and cabinet making are associated with the highest exposures (especially during machine sanding and similar operations, frequently resulting in air concentrations of wood dust greater than 5 mg/m^3^). Lower concentrations have been detected in finishing departments of plywood and particle-board mills and in the workroom air of sawmills and planer mills near chippers, saws, and planers. On the basis of the marked increase in the occurrence of SNC among woodworkers, the IARC concluded to assign wood dust to the group 1 of carcinogens to humans [24]. Sinonasal tumors have been observed also within the footwear manufacturing industry; they are probably associated with exposure to leather soles and heels dust, usually occurred in the preparation, press and finishing rooms of factories making boots and shoes by the welted process.

High relative risks of SNC have been observed also for specific chemical exposures and occupational settings, including textile industry [16,17,25-28], farming [29], construction, miners, drillers, blasters, plumbers, machinists [17,30], bakers and pastry confectioners [31], metal industry (chromium and nickel compounds) [16], and formaldehyde [23,32-36]. Textile dust has been considered a possible risk factor for SNC since certain fibers derive from plant materials (cotton, linen, rayon), and may produce exposures similar to furniture workers and cabinet makers. A key factor in textile dust carcinogenesis is likely irritation, as most mixtures related to nasal cancer in humans are aerosols (wood dust, leather dust, textile dust, chromate- and nickel-containing materials) [27]. Cancers of the nose and nasal sinuses have been reported in workers exposed to nickel compounds in nickel refining, cutlery factories, and alkaline battery manufacture, or to hexavalent chromium in chromate production and chrome plating [37]. Formaldehyde is mainly used in the manufacture of phenolic urea, melamine and acetal resins, for producing adhesives and binders for wood, plastics, textiles and leather. It is used extensively also for preparing disinfectants and preservatives and as discharge agent to the ink for printing. On the basis of sufficient evidence in humans [38,39] and in experimental animals [40,41] formaldehyde has been classified as carcinogenic for humans (IARC group 1), although with a limited evidence for SNC.

Several other occupations were found associated to SNC in case-control studies but most of them lack statistical power to identify excess risks in specific jobs [29].

A relationship between histotypes, anatomical site and occupational exposures has not been established clearly because only in few studies the onset site of the disease was exactly determined [42]. Nonetheless, several studies found higher risks of adenocarcinoma (AC) among woodworkers [43]. For other histotypes the relationship appears less consistent and the risk much lower [44].

Through a systematic review followed by a meta-analysis, this study is aimed at investigating the possible relationships between occupational exposures and SNC risk in view of suggesting opportunities for prevention.

## Methods

### Study identification

A systematic review of studies was performed by a qualitative summary of published results. We conducted a search of PubMed (http://www.ncbi.nlm.nih.gov/sites/entrez), Google Scholar (http://scholar.google.it/) and Scopus (http://www.scopus.com/) using as key words “sinonasal”, “cancer”, “occupational”, “risk”, “epidemiology”. We looked for additional studies by checking references in all identified publications. Complete articles about occupational risks for SNC were used to collect the following information: publication year (from 1968 to 2013), time period, type of publication, language, study design, topic, population studied, anatomical site, histologic subgroups, carcinogen exposure limits and criteria used to evaluate the quality of the evidence (sample size, statistical methods, measurement error, confounding and other forms of bias, statistical confidence).

Subsequently, studies were included in the meta-analysis when they complied with the following inclusion criteria:

- Articles published in peer reviewed journals;
- English language;
- Epidemiologic studies published after 1985, with a case-control or cohort design;
- Studies involving humans (men or/and women);
- Including the SNC subtypes AC and SCC;
- Referring to occupations and/or occupational setting with a potential risk of SNC;
- Exposure or potential exposure to specific risk factors stated explicitly, or from an industry/economic-activity recognized as having exposure to the risk factor (e.g. exposure to hexavalent chrome includes chromate production, stainless-steel welding, chrome pigment production, chrome plating, and ferrochrome production);
- Providing effect estimates with the corresponding measures of variability, or available data allowing for their calculation.

Finally, studies were excluded if they did not report original results (reviews, letters, comments) or did not provide sufficient data (e.g. lack of information about the number of cases and controls or about the used method).

### Data extraction

An abstract form of the most relevant available information (study type, population and location, sex, years of SNC diagnosis, type of exposure assessment, number of cases and controls in each case-control study, number of observed and expected cases in each cohort study, duration and level of exposure, risk estimates with their 95% confidence intervals for all SNC histotypes grouped together and - when available - for AC and SCC separately, covariates controlled) was created. When selected articles provided risk measures (OR/RR/SMR/SIR) stratified by specific variables (such as occupational setting, histologic subtype, duration and level of exposure), all the reported estimates were taken into account. An overview of the characteristics of the included studies can be found in Table 1, details of single studies are reported in the Additional file 1: 4th paragraph, Tables A and B.

**Table 1:** **General abstract form of the studies included in the meta-analysis**

Reference [N]	Study type	Population and location	Cases/ observed (N)	Controls/ expected (N)	Year of diagnosis	Type of exposure	Type of exposure assessment
**Pippard EC, Acheson ED 1985** [13]	cohort	5017 (3434 dead); UK, England (National Health Service Central Register)	10	1.87	up to 1982	Shoe Manufacturing	census records
**Brinton LA et al 1985** [25]	case-control	USA (North Carolina, Virginia)	160	290	1970-1980	Textile/Clothing Industries	telephone interview
**Hayes RB et al 1986** [32]	case-control	The Netherlands	91	195	1978-1981	Formaldehyde	interview
**Olsen JH, Asnaes S 1986** [33]	case-control	Denmark (Danish Cancer Registry)	466	2465	1970-1982	Formaldehyde	record linkage
**Hayes RB et al 1986** [51]	case-control	The Netherlands	116	259	1978-1981	Wood-related occupations	interview
**Merler E et al 1986** [18]	case-control	Italy (Lombardy)	20	39	1968-1982	Leather dust	interview
**Vaughan TL et al 1986** [34]	case-control	USA (western Washington state)	53	552	1979-1983	Formaldehyde	telephone/next-of-skin interview
**Fukuda K et al 1987** [53]	case-control	Japan (Hokkaido Island)	106	212	1982-1984	Carpentry/Joinery/Furniture/ Woodworking	mail questionnaire
**Roush GC et al 1987** [35]	case-control	USA (Connecticut)	198	605	1935-1975	Formaldehyde	clinical records/death certificates
**Sorahan T et al 1988** [69]	cohort	2689 (1288 M, 1401 F); UK (Office of Population Censuses and Surveys - OPCS)	3	0.3	1946-1983	Nickel/Chrome	census records
**Davies JM et al 1991** [70]	cohort	2298; UK (Office of Population Censuses and Surveys; Scottish General Register Office)	4	0.6	1950-1988	Chromate Production	census records
**Luce D et al 1992** [30]	case-control	France	207	323	1986-1988	Farming/Textile/Leather/ Woodworking/Construction	interview
**Comba P et al 1992** [16]	case-control	Italy (provinces of Verona, Vicenza and Siena)	78	254	1982-1987	Farming/Textile/Leather/ Woodworking/Construction	interview/mail questionnaire
**Comba P et al 1992** [17]	case-control	Italy (province of Brescia)	35	102	1980-1989	Farming/Textile/Woodworking/Mining/ Construction	telephone interview
**Magnani C et al 1993** [28]	case-control	Italy (Biella)	33	131	1976-1988	Woolen textile manufacturing industry	questionnaire
**Andersen A et al 1996** [66]	cohort	379 + 4385; Norway (Norwegian Cancer Registry)	32	1.8	1953-1993	soluble Nickel compounds	exposure matrix
**Fu H et al 1996** [57]	cohort	4215 Italian, 2008 English; Italy (Florence); England (Rushden, Stafford, Street)	12 *(English)* 1 *(Italian)*	0.02 *(English)* 0.001 *(Italian)*	1950-1991	Shoe Manufacturing	job title information
**Teschke K et al 1997** [72]	case-control	Canada (British Columbia Cancer Agency)	48	159	1990-1992	Farming/Textile/Paper/Leather/Forestry/ Woodworking/Construction	in person/telephone interview
**Järup L et al 1998** [67]	cohort	869; Sweden (Swedish cause of death registry, Swedish cancer registry)	3	0.36	1940-1998	Cadmium/Nickel	job exposure matrix
**Anttila A et al 1998** [68]	cohort	1388 (1339 M, 49 F); Finland (Finnish Cancer Registry)	2	0.2	1945-1985	Nickel Refinery	company's employment records
**Innos K et al 2000** [22]	cohort	6786 (3723 M, 3063 F); Estonia	3	1.6	1968-1995	Wood dust	company's employment records
**Zhu K et al 2002** [10]	case-control	USA (cancer registries)	70	1910	1984-1988	Pesticide/Chlorophenols/ Chromium compounds	telephone/next-of-skin interview
**Coggon D et al 2003** [36]	cohort	14014; British chemical factories	2	2.3	2000	Formaldehyde	company's employment records
**Hemelt M et al 2004** [21]	cohort	921 (739 M, 182 F); Swedish Family-Cancer Database	87	45	1961-2000; 1970-2000	Woodworkers	census records
**d'Errico A et al 2009** [56]	case-control	Italy (Piedmont SNC Registry)	113	336	1996-2000	Wood/Leather/Organic solvents/Welding fumes/Arsenic	questionnaire
**Mayr SI et al 2010** [74]	case-control	Germany (University of Erlangen-Nuremberg)	58	85	1973-2007	Wood/Formaldehyde	interview
**Greiser EM et al 2012** [20]	case-control	Germany (Bavaria clinical tumour registries, Baden-Wurttemberg hospitals)	427	2401	Starting 1990	Nasal stuff, smoking, hardwood dust, asbestos, organic solvents	questionnaire
**Siew SS et al 2012** [23]	cohort	1.2 million men (Finnish Cancer Registry)	32; 17	20; 15	1971-1995	Wood dust/Formaldehyde	census records

### Data analysis

#### Classification of exposures

Exposures were classified according to the HSE method [11] that is based on the IARC classification of “occupational agents, mixtures and exposure circumstances” into groups 1 and 2A with nasal cavity and parasinuses as the target organs [45]. Exposure to wood dust included logging and sawmill working, pulp and paper industry, furniture industry, cabinetmaking, joinery and carpentry, woodworking machine operating, wood manufacturing, forestry; leather dust included leather, boot and shoe industries; chromium included its alloys and compounds, chromate production, chrome bath, chrome plating; nickel included soluble nickel compounds, nickel refinery, welding, welding fumes. Other considered exposures were formaldehyde and textile industry (including tailoring, clothing, garment working). Exposures in farming and construction (not reported in the HSE classification) were included in the analyses, the former including agriculture and farm working, the latter plasterwork, mining, bricklayers, plasters and cement workers.

#### Statistical pooling

Separated analyses were performed for case-control and cohort studies, for all SNC grouped together and for each group of exposures. For case-control studies separate pooled risk estimates were calculated by the most common subtypes (AC, SCC). Other histologic types were not studied because the classifications used were not comparable between studies and no information on occupational exposures were available. When not stated, crude risk estimates and 95% confidence intervals were calculated with the reported numbers, standard deviations were calculated by their confidence intervals (exact or normal approximated) and the number of cases or controls, exposed or not exposed, was calculated starting from the risk estimate and the sample size.

In order to calculate the pooled estimate and its confidence intervals, we first used a fixed-effect model with the inverse variance weighting method [46] with pooled estimate ($\overline{T}$) equal to

$$ \overline{T}=\frac{{\displaystyle \sum_i{\overset{\wedge }{w}}_i{T}_i}}{{\displaystyle \sum_i{\overset{\wedge }{w}}_i}}, $$

where *T*_*i*_ is the log risk ratio for the *i*^*th*^ study and its weight *ŵ*_*i*_ is the inverse of variance (*1/S*_*i*_). Confidence intervals were obtained by normal approximation. When the heterogeneity hypothesis appeared relevant, the random-effect model with DerSimonian and Laird estimation method was preferred [47].

The amount of variation between the collected effect sizes is shown together with the pooled estimates by the forest plots.

#### Evaluation of heterogeneity

The *Q statistical test* was used to determine the homogeneity among the studies, with degrees of freedom equal to the number of studies minus one*.*

*I*^*2*^*index* was used to quantify the heterogeneity among studies as the percentage of the total variation not attributable to chance [48]. The contribution of each study to the total heterogeneity of the pooled data was calculated and results presented in the Additional file 1: 1st and 2nd paragraphs.

#### Subgroup analyses

When heterogeneity was present and data were able to be stratified (homogeneous strata among studies containing at least five estimates) potential sources of variability were explored through subgroup analyses. For formaldehyde we investigated the effect of exposure level (low/moderate, high) while for textile, wood and leather dust, that of exposure time (<15, ≥15 years). Although if for leather dust original exposure durations of selected studies were <15 and ≥12 years, according to the other analyses we consider as categories <15 and ≥15 years. Subgroup specific pooled relative risks were assessed and their difference was tested by the Z test.

#### Publication bias

In order to assess publication bias we used funnel plots and Egger’s test (a linear regression method, see Additional file 1: 3rd paragraph) to evaluate their asymmetry [49].

All analyses were performed with StataCorp. 2009. Stata Statistical Software: Release 11. College Station, TX: StataCorp LP.

## Results

The search in PubMed, Google Scholar and Scopus yielded more than 1,300 results but the most were excluded because regarding anatomical cancer sites other than sinonasal cavities (e.g. nasopharynx, lung, oral cavity, oropharynx, sinonasal inverted papilloma), or nasal cancer risk factors other than occupational (e.g. lifestyle) or not included in the present study (e.g. pesticides, food industry). Additional exclusion criteria were: previous studies of the same author/authors (the most recent have been chosen), books or book chapters, languages other than English, and studies without a case-control or cohort design (e.g. toxicological, case-report, molecular epidemiology studies, reviews).

**Figure 1:**
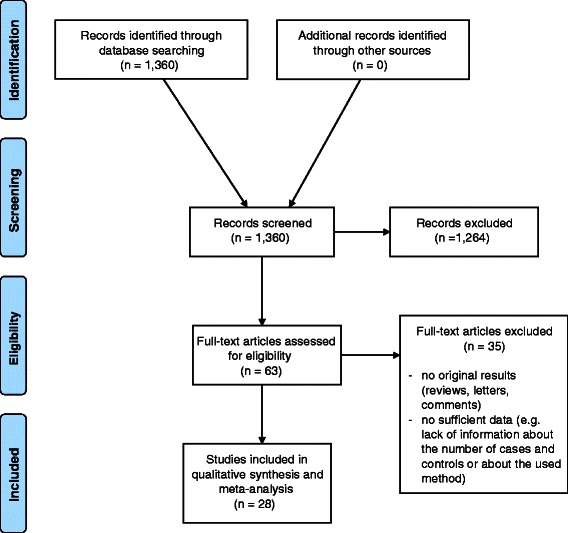
**Flow diagram of study selection.**

Residual 63 articles were reviewed and 28 out of these (11 cohort, 17 case-control) met the inclusion criteria (Figure 1), and were used in the meta-analysis (Table 1 and Additional file 1: 4th paragraph, Tables A and B).

**Figure 2:**
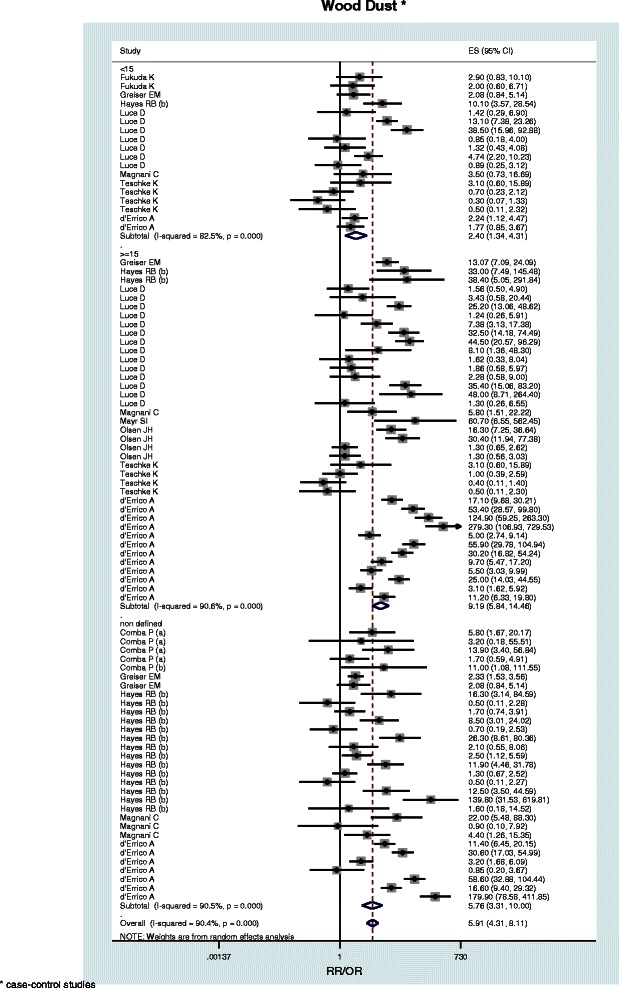
**Forest plot of study-specific RRs and RR**_**pooled**_**(95% CIs), stratified by subgroups of exposure parameters for wood dust.** The size of the squares reflects the statistical weight of the study in the meta-analyses.

**Figure 3:**
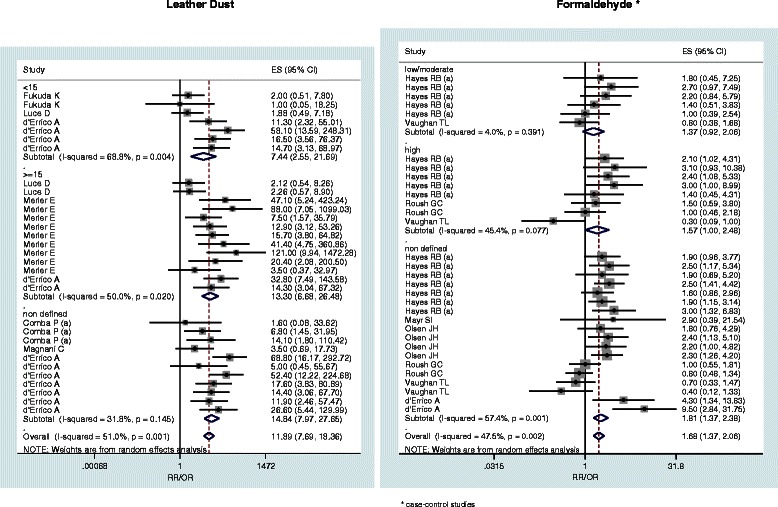
**Forest plots of study-specific RRs and RR**_**pooled**_**(95% CIs), stratified by subgroups of exposure parameters for leather dust and formaldehyde.** The size of the squares reflects the statistical weight of the study in the meta-analyses.

**Figure 4:**
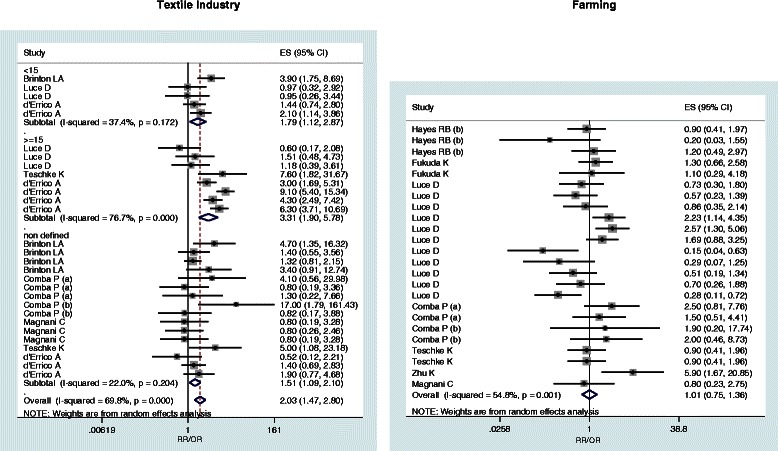
**Forest plots of study-specific RRs and RR**_**pooled**_**(95% CIs), stratified by subgroups of exposure parameters for textile industry and farming.** The size of the squares reflects the statistical weight of the study in the meta-analyses.

**Figure 5:**
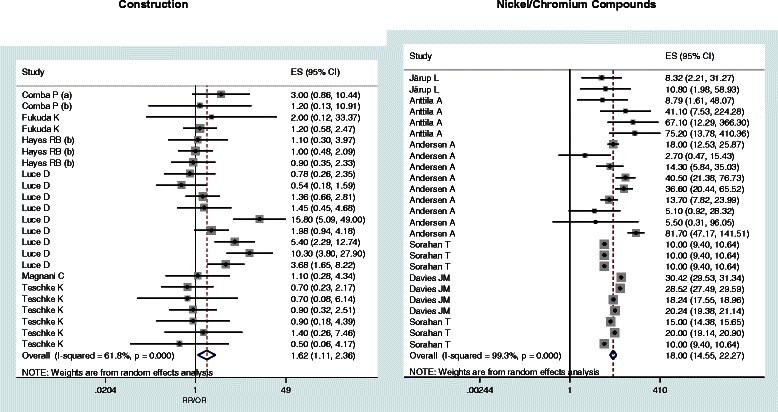
**Forest plots of study-specific RRs and RR**_**pooled**_**(95% CIs), stratified by subgroups of exposure parameters for construction and Nickel/Chromium compounds.** The size of the squares reflects the statistical weight of the study in the meta-analyses.

When available, pooled estimates (with 95% confidence limits) by AC and SCC histologic subtypes are reported (Table 2). Subgroup relative risks are reported only for wood dust, leather dust, formaldehyde and textile industry (Table 3). Figures 2, 3, 4 and 5 show the forest plots of all the study-specific risk estimates for the exposures investigated (for wood dust, leather dust and formaldehyde they are stratified by subgroups of exposure parameters). Because of the small number of estimates for cohort studies on wood dust (3) and formaldehyde (2), neither forest nor funnel plots have been performed.

**Table 2:** **Pooled estimates of SNC relative risk and 95% confidence intervals by histologic subgroup (AC, SCC) for each exposure**

	All	Adenocarcinoma (AC)	Squamous cell CA (SCC)	Exposure
**RR**_**pooled**_ (95% CI)	5.91 (4.31-8.11)	29.43 (16.46-52.61)	1.46 (1.01-2.1)	**Wood dust**
**Between studies variance** (τ^2^)	1.93	1.10	0	
**Q statistic** *(df, p-value)*	909.1 *(87, <0.01)*	81.1 (*16, <0.01*)	4.72 (*10, 0.909*)	
**I** ^**2**^	90.4	80.3	0	
**RR**_**pooled**_ (95% CI)	11.89 (7.69-18.36)	35.26 (20.62-60.28)	2.09 (1.12-3.9)	**Leather dust**
**Between studies variance** (τ^2^)	0.75	0	0	
**Q statistic** *(df, p-value)*	61.3 *(30, <0.01)*	5.18 *(9, 0.818)*	0.82 *(6, 1)*	
**I** ^**2**^	51.0	0	0	
**RR**_**pooled**_ (95% CI)	1.68 (1.37-2.06)	3.81 (1.39-10.41)	2.37 (1.69-3.33)	**Formaldehyde**
**Between studies variance** (τ^2^)	0.15	0.39	0	
**Q statistic** *(df, p-value)*	59.0 *(31, <0.01)*	4.0 *(2, 0.14)*	0.9 *(4, 0.92)*	
**I** ^**2**^	47.5	49.9	0	
**RR**_**pooled**_ (95% CI)	2.03 (1.47-2.8)	3.50 (1.88-6.54)	0.85 (0.40-1.8)	**Textile industry**
**Between studies variance** (τ^2^)	0.47	0.47	0	
**Q Statistic** *(df, p-value)*	92.8 *(28, <0.01)*	24.08 *(6, <0.01)*	0.78 *(2, 0.676)*	
**I** ^**2**^	69.8	75.1	0	
**RR**_**pooled**_ (95% CI)	1.01 (0.75-1.36)	0.38 (0.21-0.69)	1.30 (0.90-1.88)	**Farming**
**Between studies variance** (τ^2^)	0.28	0	0.07	
**Q statistic** *(df, p-value)*	50.8 *(23, <0.01)*	2.38 *(3, 0.497)*	8.6 *(6, 0.2)*	
**I** ^**2**^	54.8	0	30.2	
**RR**_**pooled**_ (95% CI)	1.62 (1.11-2.36)	0.90 (0.39-2.08)	2.15 (1.01-4.58)	**Construction**
**Between studies variance** (τ^2^)	0.48	0	0.45	
**Q statistic** *(df, p-value)*	57.6 *(22, <0.01)*	0.16 *(1, 0.691)*	11.88 *(4, 0.02)*	
**I** ^**2**^	61.8	0	66.3	
**RR**_**pooled**_ (95% CI)	18.0 (14.55-22.27)			**Nickel/Chromium compounds**
**Between studies variance** (τ^2^)	0.19	-	-	
**Q statistic** (df, p-value)	3268.0 *(24, <0.01)*			
**I** ^**2**^	99.3

**Table 3:** **Pooled estimates of SNC relative risk and 95% confidence intervals by subgroups within exposures to wood dust, leather dust, formaldehyde and textile industry (case-control studies)**

Group/subgroup	N of risk estimates/N of studies	Pooled estimate(95% CI)	p-value	Exposure
**By duration of exposure**			0.001	**Wood dust**
<15 years	18/7	2.40 (1.34-4.31)		
≥15 years	39/7	9.19 (5.84-14.46)		
**By duration of exposure**			0,371	**Leather dust**
<15 years	7/3	7.44 (2.55-21.70)		
≥15 years*	13/4	13.30 (6.68-26.48)		
**By level of exposure**			0.664	**Formaldehyde**
low	6/2	1.38 (0.92-2.06)		
moderate/high	8/3	1.57 (1.00-2.48)		
**By duration of exposure**			0.10	**Textile industry**
<15 years	5/3	1.79 (1.12-2.87)		
≥15 years	8/3	3.31 (1.90-5.78)		

*One of the included study reported risk estimates for duration of exposure ≥12 years.

In the following sections the epidemiological evidence about associations between occupational exposures and risk of SNC are summarized and the pooled risk estimates presented.

### Wood dust

A case study among wood-workers and an analysis from the Swedish Family-Cancer Database evidenced a significant high risk for SNC in all wood-related occupations [21,50]. Adenocarcinomas were strongly associated with ever being employed in furniture and cabinet making (OR = 139.8, 95% CI: 31.6-999.4), as well as in factory joinery and carpentry work (OR = 16.3, 95% CI: 2.8-85.3) [51]. However, in a Belgian retrospective case study on SNC, several dusts other than wood - including textile, cereals, cement and leather - were found in a large percentage of professional histories [52]. Men’s occupational history of being a carpenter, joiner, furniture worker, or other woodworker, resulted as risk factor of developing SCC of the maxillary sinus (RR = 2.9, p < 0.05) [53]. Among male workers exposed to levels higher than 5 mg/m^3^, an excess risk was found for AC (OR = 12.20, 95% CI: 7.43-12.20) [43]. In a case-control study in North Carolina and Virginia working in the furniture industry was associated with SNC (RR = 5.68, p < 0.05). In addition, people employed in other industries involving possible exposure to wood dust showed a risk for AC approximately 3 times higher than that for SCC (p < 0.05) [54]. A significant risk of SNC was found among Italian workers of wood and furniture industry in the province of Biella (OR = 4.4, 95% CI: 1.41-13.4) and in the province of Siena (OR = 5.4, 95% CI: 1.7-17.2), the latter with the strongest association (exposure-outcome) observed for AC (OR = 89.7, 95% CI: 19.8-407.3) [28,55]. These results were confirmed by another Italian study, where a high risk was assessed for the group including furniture makers, joiners and carpenters (OR = 6.5, 95% CI: 2.1-20), and where almost all cases of AC derived from the wood and leather industries [17]. A retrospective cohort study of furniture workers in Estonia found an increased risk for SNC, although not statistically significant (SIR = 1.87, 95% CI: 0.39-5.46) [22].

A French case-control study found significantly elevated risk of AC among male cabinetmakers (OR = 35.4; 95% CI: 18.1-69.3), carpenters, joiners (OR = 25.2, 95% CI = 14.6-43.6) and wood-working machine operators (OR = 7.4, 95% CI = 3.4-15.8). Risks associated with cabinetmakers, carpenters and joiners were also significantly elevated for the other histologic subgroups: significant excesses in risk of SCC were observed among carpenters and joiners having worked for at least 15 years in the wood manufacturing industry (OR = 8.1, 95% CI = 1.3-50.3) [30]. Elevated risk for SCC was found also in a cohort of Finnish men occupationally exposed to wood dust and formaldehyde (RR = 1.98, 95% CI: 1.19-3.31) [23].

In a case-control study in Piedmont (Italy), the occurrence of SNC was found significantly related to ever exposure to wood dust (OR = 11.4) with a risk for AC (OR = 58.6) 10-fold higher than for other histotypes. Furthermore the risk for AC doubled every 5-years of exposure period to wood dust (p < 0.0001), and significantly increased also for low intensity exposure [56]. Despite exposure effect increases with the length of its duration, an elevated risk was observed also in shorter exposure periods (less than 5 years), and evidence for a long latency times had been showed [44]. The attributable fraction (AF) of occupational exposure to wood dust was estimated at around 20% for both genders [17,43]. The most elevated values (due to high-level wood dust exposure) were observed for adenocarcinoma (75% in the population, 96% in the exposed) [51]. Exposure to hardwood dust for at least one year increased the risk for SNC (OR = 2.33, 95% CI: 1.40-3.91) in a population-based case-control study in South-Germany [20].

In the present meta-analysis on wood dust eleven case-control and three cohort studies met our inclusion criteria, contributing a total of 91 effect estimates. The pooled relative risk for SNC was estimated at 5.91 (95% CI: 4.31-8.11) for the case-control studies (Table 2) and 1.61 (95% CI: 1.10-2.37) for the cohort studies (data not tabulated). The elevated risk found for AC (RR_pooled_ = 29.43, 95% CI: 16.46-52.61) supports the findings of previous studies [16,25,28,55]. Although slight, a significant risk resulted also for SCC (RR_pooled_ = 1.46, 95% CI: 1.01-2.1). Heterogeneity among the pooled risk estimates by length of exposure time suggests a possible exposure-response relationship (RR_pooled, <15 years_ = 2.40, RR_pooled, ≥15 years_ = 9.19, p = 0.001) (Table 3).

### Leather dust

Among leather workers an increased risk of SNC (with AC as predominant) was found in an Italian study, associated with shoemaking (OR = 5.0, 95% CI: 1.9-36) [17]. Furthermore a clear dose-effect relationship had been previously observed in another Italian study, with a much stronger effect for AC in both genders [18]. Cancer risk among shoes manufacturing workers was examined in two cohorts (English and Italian), where exposures to leather dust during specific operations (scouring, roughing, buffing, spitting, skiving, cutting and trimming) were elevated. The SMR for nasal cancer was significantly high among all workers in both cohorts (however, only one case occurred in the Italian cohort). In the English cohort, most cases were reported in the manufacture of welted boots, where the presumable highest exposure to leather dust could be expected [57]. A significant dose–response relationship was found between the AC risk and exposure period to leather dust: the risk increased among workers with over 5 years’ exposure of almost 60-fold with respect to unexposed. As with wood dust, also low-intensity exposure significantly increased the risk for AC (OR = 52.4) [56]. Finally, a possible role of tannins as carcinogen agents in leather industry has been suggested [58,59]. The AF for leather dust was estimated in the range 3-13% for both genders [17,19,43].

By providing 31 effect estimates of leather dust exposure, six studies were met by inclusion criteria of our meta-analysis. The pooled relative risk for SNC was estimated at 11.89 (95% CI: 7.69-18.36) and a strong association with AC was found (RR_pooled_ = 35.26, 95% CI: 20.62-60.28) (Table 2). The exposure time effect appeared non significant (RR_pooled,<15 years_ = 7.44, RR_pooled, ≥15 years_ = 13.30, p = 0.371) (Table 3).

### Formaldehyde

A meta-analysis study found a significant association between SNC risk and formaldehyde exposure (RR = 1.75, 95% CI: 1.21-2.43) and evidenced an exposure-response gradient, although confounding by wood dust could be of concern in some of the included studies [60]. Another meta-analysis – where differences were observed between US (null results) and European studies (moderately elevated risk) – also evidenced wood dust exposure as possible confounder. Overall data from such meta-analysis do not suggest a relationship between formaldehyde exposure and SNC risk [61]. A small effect of formaldehyde on SNC could not be ruled out in a cohort of Finnish men occupationally exposed to wood dust and formaldehyde (RR = 1.1, 95% CI: 0.66-1.87) and in an extended follow-up of an existing cohort of men employed at six British factories where formaldehyde was produced or used, with two deaths (vs 2.3 expected) recorded [23,36]. No significant associations were found in a population-based case-control study in western Washington with any level or number of years of exposure [34]. An association with SNC was found among printers (RR = 1.1, 95% CI: 0.4-3.2) in a case-control study in Connecticut (US) [35]. A broad review of cancer in industry workers and professionals who used formaldehyde, such as pathologists, anatomists and embalmers, evidenced no significant excess risk for SNC [62]. A pooled analysis on SNC and occupational exposures found an exposure-risk gradient in both genders even after a check for residual confounding (wood dust) [63]. In a previous study, an association between SNC risk and formaldehyde exposure was observed (RR = 2.8; 95% CI: 1.8-4.3) that reduced after adjustment for wood dust, in accordance with an additive effect [64]. An increased risk for SNC was found in a large-scale Danish study (Standardized Proportionate Incidence Ratio - SPIR = 2.3, 95% CI: 1.3-4.0), with elevated risk among workers exposed to both wood-dust and formaldehyde (SPIR = 5.0, 95% CI: 0.5-13.4), and among moderately exposed to formaldehyde, but probably not to wood dust (SPIR = 3.0; 95% CI: 1.4-5.7). In this study exposure to wood dust does not appear a major confounder [65].

The estimated total (male and female) AF for SNC associated with occupational exposure to formaldehyde is 0.17% (95%CI = 0.10-0.45) [11].

Results of our meta-analysis were based on six case-control and two cohort studies, providing a total of 34 effect estimates. The pooled SNC relative risk was 1.68 (95% CI: 1.37-2.06) for the case control (Table 2) and 1.09 (95% CI: 0.66-1.79) for the cohort studies (data not tabulated). A modest increased risk was observed among low (RR_pooled, low_ = 1.38) and moderate/high level of exposure (RR_pooled,__moderate/high_ =1.57), with non significant difference (p = 0.664) (Table 3).

### Nickel and chromium

An increased risk of SNC was found in nickel refinery workers (SIR = 18, 95% CI: 12-25); in workers with the highest level of nickel exposure (≥15 mg/m^3^) the risk for SNC was higher than that for lung cancer, with a dose-response gradient for both nickel oxide and soluble nickel [66]. Association between exposure to nickel/cadmium and risk for SNC was investigated in a cohort of Swedish battery workers, where a strong risk was found in men (SIR = 832, 95% CI: 172-2430) [67]. Nasal cancer incidence has been studied among workers at a Finnish copper/nickel smelter and nickel refinery where the risk resulted significant (SIR = 41.1, 95% CI: 4.97 - 148) and increased with the duration of employment (SIR = 75.2, 95% CI: 9.10-271) [68]. Exposure to chromium in the form of chromic acid mist (chromium oxide, CrO_3_ - soluble in water, hexavalent chromium) was investigated in a group of chrome platers in UK, with a significant excess of occurrences (p < 0.05) observed in several death causes, including SNC (2 observed cases in men versus 0.2 expected, employed in chrome bath work) [69]. Similarly, a study regarding three UK chromate producing factories found significant excess of mortality from nasal cancer (SMR = 1538). All the four affected men had over 20 years of employment [70]. The total AF for SNC associated with hexavalent chromium was found at 5.7% [71].

In the present meta-analysis six studies about Nickel/Chromium exposure met our inclusion criteria, contributing a total of 25 effect estimates. The pooled relative SNC risk was 18.0 (95% CI: 14.55-22.27) (Table 2).

### Other exposures (textile industry, construction, farming)

Among female textile workers, a significant increase in risk of SCC (OR = 9.5, 95% CI = 1.7-54.1) and a moderate increase in risk of AC (OR = 4.0, 95% CI = 0.7-23.5) was observed in a case-control study in France [30]. An increased risk of SNC for textile workers (OR = 7.6, 95% CI = 1.4-56.6) has been identified also in a surveillance follow-up in British Columbia (Canada) [72]. No association was found in an Italian case-control study in a large woolen textile industry (OR = 0.8, 95% CI: 0.2-2.8) [28].

In France, significant deaths excess of SCC were observed for construction workers (OR = 3.7, 95% CI = 1.7-8.0) and for farm workers of both sexes (males: OR = 2.2, 95% CI = 1.1-4.4; females: OR = 4.9, 95% CI = 1.0-24.9) [30].

The pooled relative risk for exposure in the textile industry from all 29 risk estimates of the six selected articles in this study, evidenced a significant risk of all SNC (RR_pooled_ = 2.03, 95% CI: 1.47-2.8) which increased for AC (RR_pooled_ = 3.5, 95% CI: 1.88-6.54), with significant heterogeneity (Table 3). Stratification by exposure time showed a non-significant higher risk for a longer duration (RR_pooled,<15 years_ = 1.79, RR_≥15 years_ = 3.31, p = 0.10).

In the construction sector (including plasterwork, mining, bricklayers, plasters and cement workers) a significant pooled risk has been observed (RR_pooled_ = 1.62, 95% CI: 1.11-2.36), while farming resulted non significantly associated with the risk of SNC (RR_pooled_ = 1.01, 95% CI: 0.75-1.38) neither with SCC (RR_pooled_ = 1.30, 95% CI: 0.90-1.88).

### Heterogeneity and publication bias

**Figure 6:**
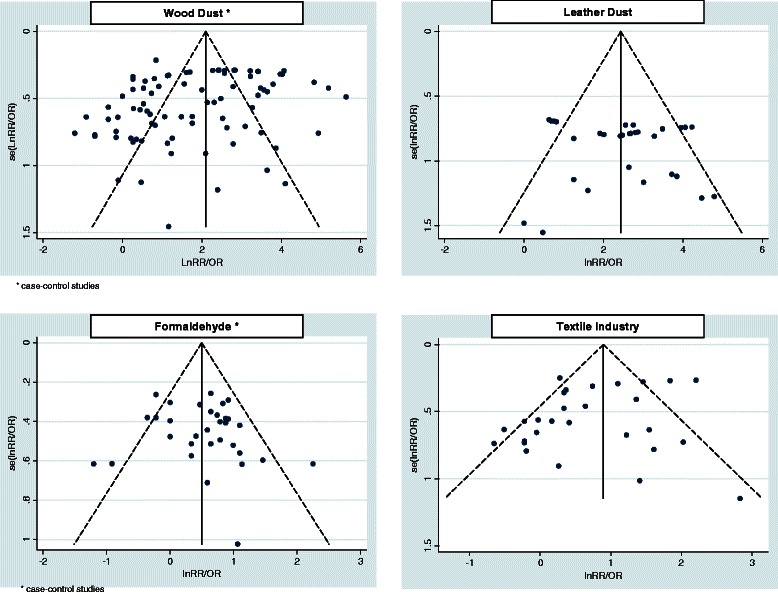
**Funnel plots of studies included in the meta-analysis for the risk of SNC associated with exposure to wood dust, leather dust, formaldehyde and textile industry.** Note: Funnel plots can be easily interpreted by including diagonal lines representing the 95% confidence limits around the pooled estimate, to show the expected distribution of studies in the absence of heterogeneity or of selection bias: in the absence of heterogeneity, 95% of the studies should lie within the funnel defined by these straight lines.

**Figure 7:**
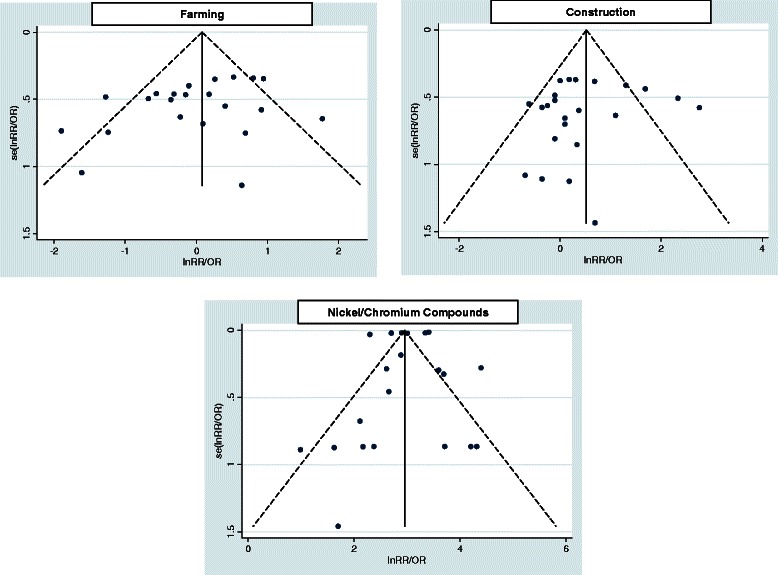
**Funnel plots of studies included in the meta-analysis for the risk of SNC associated with exposure to farming, construction and Nickel/Chromium compounds.** Note: Funnel plots can be easily interpreted by including diagonal lines representing the 95% confidence limits around the pooled estimate, to show the expected distribution of studies in the absence of heterogeneity or of selection bias: in the absence of heterogeneity, 95% of the studies should lie within the funnel defined by these straight lines.

For the selected types of exposure, heterogeneity among studies was observed and random-effects models were used. Subset analyses identified several sources of heterogeneity (Table 2). To the highest levels of exposure times and magnitudes always correspond the highest values of risks (Table 3) but this association was considered significant only for wood dust (p = 0.001). Evidence of publication bias was assessed for wood dust (Egger’s test bias = 0.009), but not for the other exposures (Egger’s test bias >0.05) (Figures 6 and 7).

## Discussion

The occurrence of SNC was significantly associated to exposures to wood dust, leather dust, formaldehyde, nickel/chromium compounds, organic solvents, welding fumes and arsenic.

Numerous cohort, case-referent and case reports studies of SNC show increased risks among workers exposed to wood dust. Moreover, in a large data collection of SNC cases with occupational exposure, a high prevalence of TP53 mutation-positive has been observed [73]. While adenocarcinomas are extremely rare in the general population, very high relative risks have been observed among workers exposed to wood dust. Occupational exposures in woodworking, such as to beech and oak wood dusts, are considered risk factors for this tumour [74,75]. The ethmoid resulted the sinonasal site mostly affected by AC [74,76]. The percentage of AC in men was found 3-4 times higher than in women but different magnitudes of exposures (exposure to wood dust often involves men) presumably affected results [43]. The other histologic subgroup of SNC associated to occupational exposures is the squamous cell carcinoma. In some studies, wood dust exposure seems to affect the risk of SCC only for women, but this finding could be probably attributable to differences in exposure to chemical agents in the workplace [44]. However, in a Japanese case-control study men’s occupational history was found a significant risk factor for SCC of the maxillary sinus (RR = 2.9) [53].

The effect of other established occupational factors on different anatomical sites has yet to be properly defined, and the etiological role of occupational risk factors in SNC different from AC and SCC needs to be better quantified. A recent study on the prevalence of occupational hazards in different SNC histologic types observed a large proportion of AC cases with documented exposure to wood and leather dust while other histotypes showed a lower proportion of cases exposed to occupational hazards [42]. In the USA the majority (59%) of sinonasal cancers diagnosed between 2004 and 2008 were epithelial neoplasms, with SCC and AC being the most common subtypes, accounting for 38% and 10% of all sinonasal cancers, respectively [77]. In the present meta-analysis 27% of SNC are represented by AC and 48% by SCC.

Sinonasal cancers are rare, but when focusing on specific histologic subgroups (e.g. adenocarcinomas) and occupational exposures, the incidence rate rise exponentially. Due to the biunivocal and strong relationship between incidence and exposure to specific carcinogens, SNC is recognized as an “occupational tumor” together with malignant mesothelioma, supporting the development of specific epidemiological surveillance systems [78].

Results from our meta-analysis indicate that SNC is related to wood dust exposure with a substantial heterogeneity among individual study estimates. Summary estimates also reveal positive associations between SNC risk and exposures to leather dust, formaldehyde, nickel/chromium compounds and in textile and construction industries.

Currently, review articles and meta-analyses for assessing small risks with large public interest or important implications for public health have been increasing, though the use of meta-analysis of observational epidemiological studies draws less consent than in the area of clinical trials. The main concern relates to the synthetic approach of meta-analysis that emphasizes summarizing evidence over the search for heterogeneity [79]. In our meta-analysis presumable confounders were investigated through stratification on several factors, with a reduction of heterogeneity. Sub-meta-analyses by duration/level of exposure have been performed. Adenocarcinoma is confirmed as the histologic subgroup mostly associated with wood/leather dust exposure, but our results show a significant association also with formaldehyde and textile industry. Stratifying by duration of exposure, the pooled relative risk resulted statistically significant for the longer durations only for wood dust exposure.

A moderate/high level of exposure to formaldehyde shows a higher risk of SNC risk than lower levels of exposures, but not statistically significant. Analyses on farming exposure did not provide any significant risk for SNC (p > 0.05), while an increased risk for SCC was found in the construction sector. Evidence of publication bias was detected only for wood dust exposure, where the regression method indicated minor risks for smaller studies.

Limitations of this meta-analysis derive from the specific characteristics of the included studies and from the general ones of the used analysis. Firstly, a greater number of studies and risk estimates have been published on wood dust exposures with respect to other exposures, thus the pooled risk estimates are affected by different sizes. Secondly, the etiologic role of the exposure to carcinogens in different histological subtypes is still disputed and grouping together all SNC types could have reduced the causal role of occupational exposure. Anyway, the most of studies included in this meta-analysis focuses on AC and SCC as the most occupation-related subtypes (all the other histotypes represent around 20% of the total, and no information about occupational exposures for these cases could be retrieved). This is why analyses have been performed on the two subtypes separately and, for a rare disease such as sinonasal cancer, this increased the power to identify risk factors or to confirm previously suggested associations. Thirdly, differences across genders were not investigated, because only 5 case-control and one cohort studies showed separate results. Fourthly, studies used different exposure assessment, implying possible misclassifications. Finally, we have introduced a classification bias by considering the duration of exposure “≥12 years” as “≥15 years” in the subgroup analysis of leather dust.

Although many studies assessed attributable fraction of SNC risk as reducing [71,80,81] some others provided risen estimates [82,83]. Controlling exposure to inhalable substances implies the elimination from the workplace air, through substitution, work in a closed circuit, modification of work methods, isolation and local ventilation, and, in some cases, by using personal protective equipment. In all activities involving dusts exposure, prevention measures should be improved and air quality controls imposed, either by containment dust transmission throughout the work environment, or by using general or local exhaust ventilation to remove the dusty air. The use of personal protective equipment should also be considered, because breathable dust is often invisible, and there may be a false sense of security about the apparent lack of emissions from processes [84].

Efforts should be made to improve informative campaigns and periodical medical checking because the anatomical site and the long latency often lead to SNC diagnosis only in advanced phases of the disease, while first tumour stages have a far better prognosis [74].

The carcinogenic potential of some occupational hazard has been definitely established in several well-conducted epidemiological studies [12,38,85-87]. Nevertheless, a complete and multidisciplinary occupational evaluation of all SNC cases should be improved, to properly highlight any possible relevant exposure to occupational hazards less common than wood and leather dusts and in all different SNC subtypes. Therefore, future investigations should focus not only on the nature of exposures but also on disease characteristics (such as histological types and precursor lesions) to support in identifying possible carcinogens and mechanisms of action.

## Conclusions

By supporting the previously reported associations between occupational exposures and SNC incidence rate (often specific for histologic subgroup, such as wood dust and adenocarcinoma), our results may provide clues to the etiology of SNC.

Our overall summary risk estimates strongly suggest that exposure to wood and leather dusts, formaldehyde, in the textile industry and to nickel/chromium compounds, increases the risk of developing SNC. A strength of this meta-analysis is that, by providing pooled SNC risk estimates, we have focused on this type of occupational cancer, that was scarcely emphasized by previous studies, mainly due to the small numbers of cases in studies.

The failure to recognize SNC as an occupational disease may imply inadequate knowledge of SNC - a serious form of cancer with high levels of mortality, that significantly compromises quality of life - and incomplete preventive measures. Therefore, greater awareness should be required in exploring the occupational etiology of SNC, in medical monitoring, in implementing new technical solutions and discussing occupational threshold exposure levels. Finally the need to implement specific epidemiological surveillance system for occupational cancers must be considered.

## Additional file

Additional file 1:
**Appendix to the manuscript including deepening sections on the heterogeneity, publication bias and details of single studies of meta-analysis.**

## Abbreviations

SNC: Sinonasal cancer
AC: Adenocarcinoma
SCC: Squamous cell carcinoma
AF: Attributable fraction
OR: Odds ratio
RR: Relative risk
SMR: Standardized mortality ratio
SIR: Standardized incidence ratio
