# Supplementary material for: Occupational exposure and sinonasal cancer: a systematic review and meta-analysis
Source: BMC Cancer. 2015 Feb 13;15:49. doi: 10.1186/s12885-015-1042-2 (PMC4339645; doi:10.1186/s12885-015-1042-2)
Supplement: Additional file 1: — Appendix to the manuscript including deepening sections on the heterogeneity, publication bias and details of single studies of meta-analysis. [file 12885_2015_1042_MOESM1_ESM.doc]

**Appendix**

**1. Z-statistic**

The contribution of each study to the total heterogeneity of the pooled data was calculated through a Z-statistic for each study in the meta-analysis. This contribution is determined by two components: the first is the difference between individual risk estimates and the pooled estimate; the second component is the study size. Studies reporting risk estimates lower than the pooled estimate will have a negative Z-score, the others a positive one. Larger studies with extreme deviations from the pooled estimate will have a greater effect on the pooled analysis results and will therefore have larger Z-scores. This information allows us to interpret the representativeness of individual epidemiologic studies in the context of data totality, as well within surrogate exposure categories. The following equation was used:

**z_i_ = (T_i_ – Ť) / [SE(T_i_)]**

where:

z_i_ = z-score for the i^th^ study

T_i_ = risk ratio for the i^th^ study

Ť = pooled estimate for the true effect value

[SE(T_i_)] = standard error of T_i_

For this meta-analysis z-scores are reported for exposures associated with exposure to wood dust, leather dust, formaldehyde, textile industry, farming, construction (case-control studies) and Nickel/Chromium compounds (cohort studies).

**a) Exposure to Wood dust**

1. **Exposure to Leather dust**

1. **Exposure to Formaldehyde**

1. **Exposure to Nickel/Chromium compounds**

**pooled estimate = 18**

1. **Exposure to Textile industry**

1. **Exposure to Farming**

1. **Exposure to Construction**

**2. Influence plots**

Influence plots combine Z-scores with study weights (elevated to 0.5) and provide a graphical representation of relative heterogeneity due to each study. They are reported for exposures associated with exposure to wood dust, leather dust, formaldehyde, textile industry, farming, construction and Nickel/Chromium compounds.


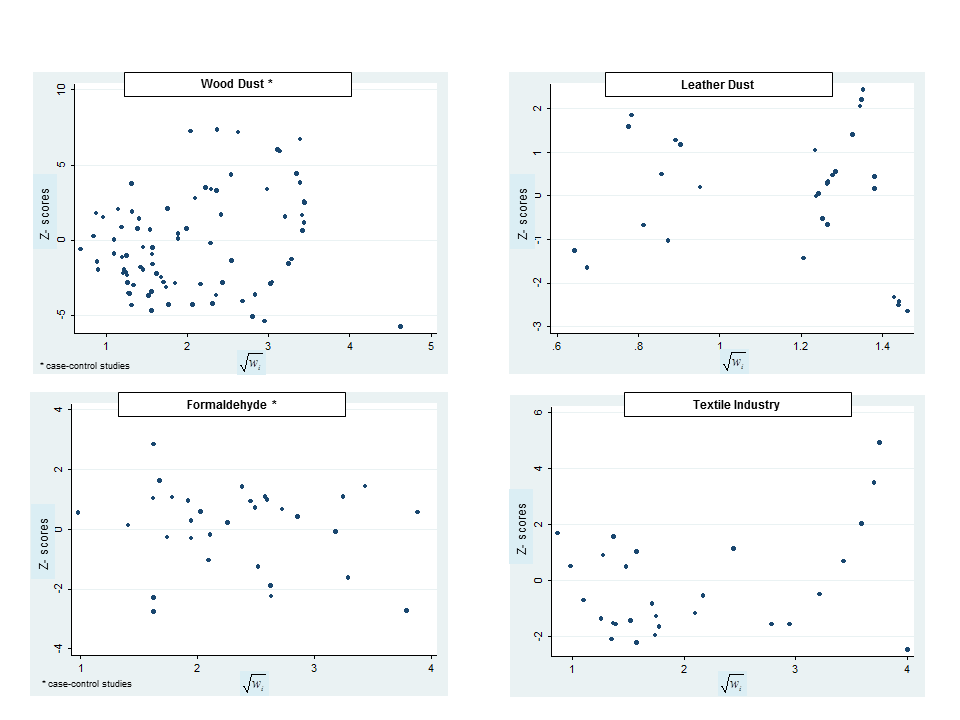


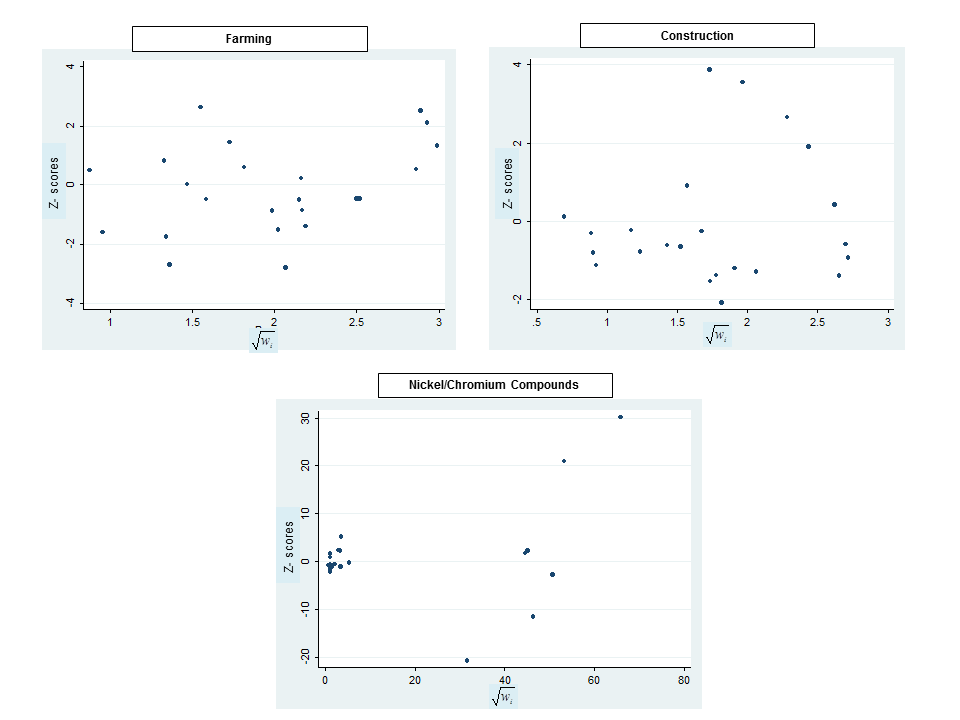


**3. Egger’s test**

In a meta-analysis publication bias is related to differential inclusion of studies whose results are significant, interesting, from large well-funded studies, or of higher quality, that are more likely to be published than the others. The resulting bias may invalidate the conclusions. If no publication bias is present the plot is shaped like a funnel. Results from small studies distribute widely at the bottom of the graph, with the spread restricting among larger studies. This is expected because studies of smaller size have increasingly large variation in the estimates of their effect size, as random variation becomes increasingly significant. Conversely, when publication bias is present - since smaller or non­significant studies are less likely to be published - studies in the bottom left hand corner of the plot are often missing, creating a degree of asymmetry in the funnel. In the linear regression method used to measure funnel plot asymmetry [49], the standard normal deviate (SND), defined as the log risk ratio divided by its standard error, is regressed against the estimate's precision, the latter being defined as the inverse of the standard error. The regression equation (SND = a + b x precision) applied specifically to the present meta-analysis, becomes:

**[lnRR_i_/SE(lnRR_i_)] = a + b x [1/SE(lnRR_i_)]**

The intercept provides a measure of asymmetry: the larger its deviation from zero, the more pronounced the asymmetry. Evidence of asymmetry is based on p-value <0.1 and intercepts are reported with 90% confidence intervals This choice depends on the generally small number of studies included in the meta-analysis, that limits the statistical power of the test.

**4. Details of single studies included in the meta-analysis**

Once the full text papers of the selected studies for the meta-analysis were available, an abstract form was created of the most relevant information provided (number of cases/controls and observed/expected in each study, relative risk estimates with their 95% confidence intervals, histology, duration and level of exposure, covariates controlled, notes). The results of this work were tabulated in a data extraction form. The following tables (A, B) report authors estimates of risk ratio and study properties for selected publications relating to occupational exposures and sinonasal cancer. Some of these studies provided independent data according to the type and level of exposure (duration and level of exposure), type of occupational setting, subtype of cancer and sex of the studied population.

**Table A. Case-control studies (N=17) included in the meta-analysis**

**Table A. (continued)**

**Table A. (continued)**

**Table A. (continued)**

**Table B. Cohort studies (N=11) included in the meta-analysis**
